# Clinical and cost evaluation of two models of specialist intensive support teams for adults with intellectual disabilities who display behaviours that challenge: the IST-ID mixed-methods study

**DOI:** 10.1192/bjo.2023.74

**Published:** 2023-06-26

**Authors:** Angela Hassiotis, Athanasia Kouroupa, Leila Hamza, Louise Marston, Renee Romeo, Nahel Yaziji, Ian Hall, Peter E. Langdon, Ken Courtenay, Laurence Taggart, Nicola Morant, Vicky Crossey, Brynmor Lloyd-Evans

**Affiliations:** Division of Psychiatry, University College London, UK; Assessment and Intervention Team, Barnet, Enfield and Haringey Mental Health NHS Trust, UK; Department of Primary Care and Population Health, University College London, UK; Institute of Psychiatry, Psychology and Neuroscience, King's College London, UK; Hackney Integrated Learning Disability Service, East London NHS Foundation Trust, UK; Centre for Educational Development, Appraisal and Research, University of Warwick, UK; Institute of Nursing and Health Research, Ulster University, Northern Ireland; South West Community Learning Disability Team & Mental Health Intensive Support and Treatment Team, NHS Lothian, UK

**Keywords:** Intellectual disability, developmental disorders, cost-effectiveness, outcome studies, intensive support

## Abstract

**Background:**

Intensive support teams (ISTs) are recommended for individuals with intellectual disabilities who display behaviours that challenge. However, there is currently little evidence about the clinical and cost-effectiveness of IST models operating in England.

**Aims:**

To investigate the clinical and cost-effectiveness of IST models.

**Method:**

We carried out a cohort study to evaluate the clinical and cost-effectiveness of two previously identified IST models (independent and enhanced) in England. Adult participants (*n* = 226) from 21 ISTs (ten independent and 11 enhanced) were enrolled. The primary outcome was change in challenging behaviour between baseline and 9 months as measured by the Aberrant Behaviour Checklist-Community version 2.

**Results:**

We found no statistically significant differences between models for the primary outcome (adjusted *β* = 4.27; 95% CI −6.34 to 14.87; *P* = 0.430) or any secondary outcomes. Quality-adjusted life-years (0.0158; 95% CI: −0.0088 to 0.0508) and costs (£3409.95; 95% CI −£9957.92 to £4039.89) of the two models were comparable.

**Conclusions:**

The study provides evidence that both models were associated with clinical improvement for similar costs at follow-up. We recommend that the choice of service model should rest with local services. Further research should investigate the critical components of IST care to inform the development of fidelity criteria, and policy makers should consider whether roll out of such teams should be mandated.

Approximately 18% of adults with intellectual disabilities (lifelong limitations in adaptive functioning evident in early life) living in the community display aggression, self-injury, property destruction or other socially inappropriate behaviours (e.g. sexual disinhibition, screaming or hitting out, etc.) in their lifetime.^1,2^ Some 24 000 adults with intellectual disabilities are at risk of being admitted to specialist psychiatric assessment and treatment units, often because of the display of such behaviours.^2,3^

Research suggests that these individuals are subject to unnecessary long-term psychotropic medication use, poorer health, abuse and social exclusion.^1,4^ International studies indicate that adults with intellectual disabilities are more likely to visit the emergency department for psychiatric issues,^5^ return to the emergency department within 30 days of discharge,^6^ be in long-term in-patient care and experience premature mortality.^7^ Failure to effectively address behaviours that challenge before a crisis arises causes significant distress and burden to families and consequent breakdown of placements,^8,9^ in addition to significant healthcare and societal costs. A recent census of the Transforming Care Programme^10^ in England, a national initiative to drive improvements in the care of people with intellectual disabilities who display behaviours that challenge, indicated minimal change in relation to the number of in-patient admissions, length of hospital stay, out-of-area placements and antipsychotic medication use, confirming concerns about the lack of progress in the care of this population group across the country.^11^ Intensive support teams (ISTs) are community services that complement the community intellectual disability services and have been in operation since the early days of community care.^12,13^ However, there is little evidence to recommend a preferred IST model, and there are no nationally specified outcomes for IST care. The National Institute for Health and Care Excellence (NICE)^14^ recognised the importance of such specialist treatment services, but did not find sufficient evidence that they were clinically effective or that they reduced costs. Hassiotis et al have reported the typology of ISTs, which led to the identification of two models, independent and enhanced.^15^ The aim of the present study was to examine the clinical and cost-effectiveness of the two IST models at 9 months follow-up.

## Method

### Study design

The primary and secondary outcomes were collected at baseline and 9 months follow-up. At the time of study completion, there were UK-wide public health measures implemented because of the COVID-19 pandemic, and in-person assessments could not take place between March 2020 and January 2021.

### Service and participant recruitment

The research team prepared a matrix of all identified IST services in England stratified by model type, case-load size and area. The service managers of ISTs representing the two models were randomly invited to take part in the study. If they refused or did not respond, the next service in the matrix was approached until the required number of ISTs and participants were enrolled. The study inclusion criteria for services were as follows: ISTs operational for at least a year and ISTs funded for the duration of the study; for patient participants, the inclusion criteria were as follows: adults aged 18 years or over with a clinical diagnosis of mild to profound intellectual disabilities, and being under the care of an IST (either model) including new referrals. Those with a primary diagnosis of personality disorder or substance misuse, or a clinical decision that taking part in the study would be inappropriate because of risks, were excluded. Potential participants and their family/paid carers were approached by researchers and, where available, staff from the Clinical Research Networks to seek expressions of interest to take part in the study.

### Consent statement

Participants provided written and/or audio-recorded verbal consent for in person or online assessments, respectively. For participants with intellectual disabilities who did not have capacity to make an informed decision about taking part in this study, we obtained written and/or audio-recorded agreement from a personal/nominated consultee.

### Ethics statement

The authors assert that all procedures contributing to this work comply with the ethical standards of the relevant national and institutional committees on human experimentation and with the Helsinki Declaration of 1975, as revised in 2008. All procedures involving human patients were approved by the London Bromley Research Ethics Committee (approval number 18/LO/0890). The study was registered with ClinicalTrials.gov (identifier NCT03586375), the Integrated Research Application System (identifier 239820) and the National Institute for Health Research (NIHR) Central Portfolio Management System (identifier 38554).

### Outcomes

The primary outcome was change in challenging behaviour as measured by the carer-reported Aberrant Behaviour Checklist-Community version 2 (ABC-C).^16^ Secondary outcomes were mental health comorbidity (Psychiatric Assessment Schedule for Adults with Developmental Disabilities Checklist; PAS-ADD Checklist),^17^ clinical risk (Threshold Assessment Grid; TAG)^18^ and quality of life (Quality of Life Questionnaire; QoL-Q).^19^ The ABC-C, PAS-ADD and QoL-Q have been validated for use with people with intellectual disabilities. The TAG is widely used in clinical practice to capture clinical risk in patients with mental illness, and has been used previously in a population with intellectual disabilities.^20^ Quality-adjusted life-years (QALYs) were derived from the EQ-5D-5L^21^ scores. If the participant with intellectual disability had sufficient reading ability, a researcher aided completion of a self-report version of the EQ-5D-5L. It is recommended that the proxy EQ-5D-5L should also be completed for adults with intellectual disabilities. Use of hospital and community services was obtained with the study-adapted Client Service Receipt Inventory,^22^ covering the previous 6 months. At 9 months follow-up, service use for the previous 6 months was extrapolated to 9 months.

### Additional information

We also collected sociodemographic details, Adaptive Behaviour Scale – Short-Form (SABS)^23^ score as proxy of intellectual disability (higher scores indicate mild intellectual disability), medication use, number of hospital admissions and changes in accommodation.

### Sample size

The sample size was calculated to detect a difference of 0.45 s.d. in primary outcome score. Assuming two IST models, this required 96 participants per group (192 in total) with 5% significance (two-sided), 80% power and an intraclass correlation coefficient of 0.02.^24^ After inflation for 15% loss to follow-up, the estimated sample size was 113 participants per model (226 participants in total).

### Statistical analyses

A detailed statistical plan was developed *a priori* and reviewed by the oversight Study Steering Committee. All analyses were carried out with Stata/IC version 16.0 for Windows.^25^ All hypothesis testing was conducted with a two-sided significance level of 5%, with corresponding 95% confidence intervals.

### Clinical effectiveness

The primary outcome was estimated with a mixed-effects linear regression model, with change in ABC-C score as the outcome, a fixed effect for IST type as the main exposure and a random effect for IST to account for clustering within services. We carried out unadjusted modelling, then age, gender, accommodation type, level of intellectual disability (SABS score), level of risk (baseline TAG score), presence of autism and/or attention-deficit hyperactivity disorder, number of physical comorbidities and presence of organic, affective and psychotic disorders (as determined by the PAS-ADD) were identified as potential confounders and were included in an adjusted model. Continuous secondary outcomes were analysed with statistical models analogous to those for the primary outcome. Binary outcomes were analysed with mixed-effects logistic regression models and were unadjusted. Analyses of secondary outcomes were considered exploratory. Predictors of missingness of the primary outcome were examined with mixed-effects logistic regression. Where there were up to 20% missing items for the ABC-C, TAG and QOL-Q, they were replaced by the mean score of items present. Where items were missing for the PAS-ADD, they were replaced by a code indicating the participant was negative for the given condition.

### Health economic analysis

A detailed health economic analysis plan was also developed *a priori* and reviewed by the oversight Study Steering Committee, and followed similar principles to the statistical analysis plan regarding assumptions. All analyses were carried out with Stata/IC version 16.0.^25^

### Economic evaluation

#### Perspective

The cost-effectiveness analysis adopted the perspectives of health and social care, which covers hospital and community health, social care service and voluntary support provided by not-for-profit organisations. Wider societal perspective also includes the cost of unpaid support to the participant by family and friends.

#### Valuation of resource use

Costs of the IST service models were derived by combining data on annual salary, working time, overheads, number of sessions with participants, information on case-loads and referrals over 12 months. Travel costs to home visits were included where this was noted. The annual cost was then weighted to derive a cost per study participant for each IST model over 9 months. Unpaid support costs were calculated with the market price approach, the hourly rate of a home care worker was used for those not in employment and if employed, the carer hourly wage rate. All unit costs were for the financial year 2020/2021.

#### Cost-effectiveness

We analysed differences in mean health and social care costs and wider societal costs at 9 months in turn between the IST models, by regressing total cost from each perspective on IST model, baseline costs, total ABC-C score, health-related quality of life tariffs and a range of clinical and sociodemographic indicators. Non-parametric bootstrapping was used to estimate 95% confidence intervals for mean costs. Significance was set at *P* < 0.05.

Cost-effectiveness was explored with the net benefit approach,^26,27^ with effectiveness measured in terms of the primary outcome measure (ABC-C score), and QALY gains were derived by developing value sets from the EQ-5D-5L by means of a cross-walk to the EQ-5D-3L value sets^28^ at each time point. Uncertainty around the cost and effectiveness estimates was represented by cost-effectiveness acceptability curves.^29^

In sensitivity analyses, we examined whether adjustment for baseline characteristics affected the main findings. Those variables identified as significantly associated with missingness were then added to the baseline covariates used in main analyses and new incremental cost-effectiveness ratios were re-estimated.

### COVID-19 impact and adaptations

Three National Health Service (NHS) sites withdrew their participation when the NIHR suspended all non-COVID-19-related research in March 2020. To carry on with recruitment, we applied for and received ethical approval to complete the consent process and research assessments remotely, using digital platforms (e.g. Zoom, telephone calls, scanned copies via email). Challenges to the study included digital poverty (e.g. lack of computer/smartphone), insufficient knowledge of using digital platforms and where a participant could receive support from if doing so, difficulty in assessing whether a patient with intellectual disabilities had sufficient verbal ability to provide consent remotely and delays in obtaining contact details for consultees.

## Results

### Clinical outcomes

**Fig. 1:**
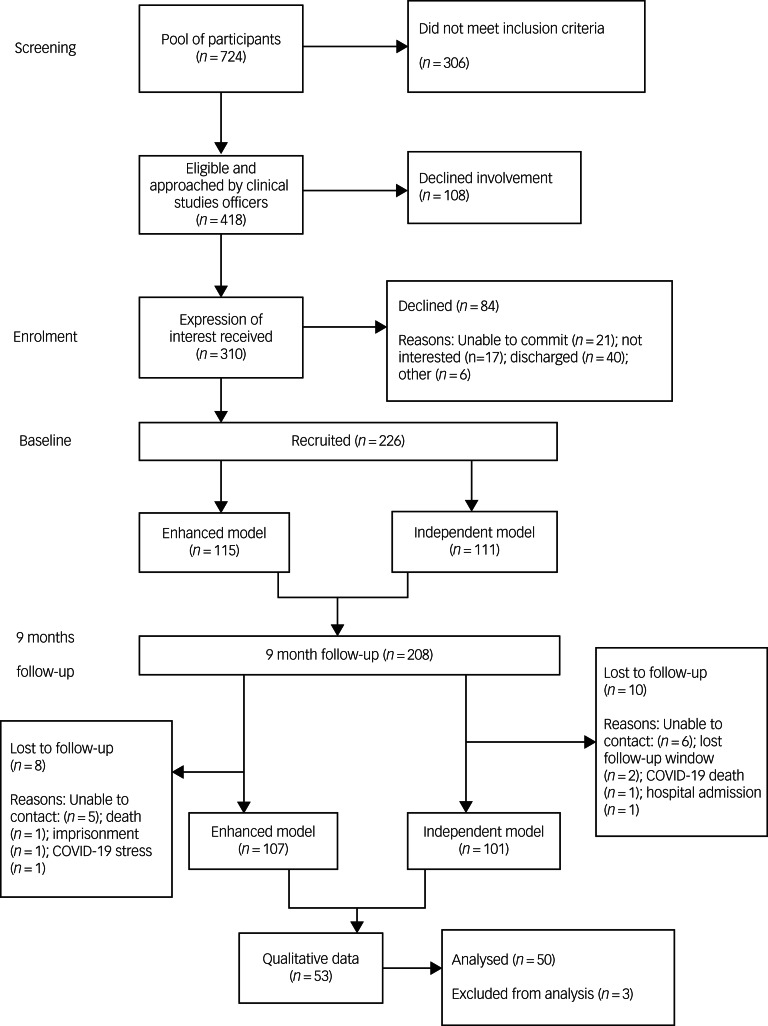
STrengthening the Reporting of OBservational studies in Epidemiology (STROBE) diagram.

The STrengthening the Reporting of OBservational studies in Epidemiology (STROBE) diagram (Fig. 1) presents the participant flow into the study. Enrolment took place between September 2018 and May 2020, with the last participant assessment in January 2021. There was an 8% attrition rate because of the following reasons: uncontactable (*n* = 12), death (*n* = 2, of which one was because of COVID-19), missing follow-up assessment window (*n* = 2), imprisonment (*n* = 1) and excessive stress during the pandemic (*n* = 1).

Demographic characteristics of adults with intellectual disabilities per IST model at baseline and 9-month follow-up are presented in Table 1. The median age of participants was 29 years old (interquartile range (IQR) 23–39) and the majority were single male of White ethnicity. More than 60% of participants had comorbid developmental disorders. The whole cohort level of adaptive ability was 52 (s.d. = 24). Participants in the two models differed in the number of reported hearing or visual problems (enhanced 52% *v*. independent 68%; *P* = 0.018) and education status (enhanced 45% *v*. independent 32%; *P* = 0.035). At follow-up, participants were more likely to be receiving care from the enhanced IST compared with those still in contact with the independent IST (enhanced *n* = 78, 74% *v*. independent *n* = 45, 45%; *P* = 0.001). The median time adults with intellectual disabilities were seen from the enhanced ISTs was 20 months (IQR = 12–33) compared with 13 months in independent ISTs IQR = 10–22).

**Table 1:** Baseline sociodemographic and clinical characteristics by intensive support team model

	Baseline		
	Enhanced (*n* = 115), mean (s.d.) or *n* (%)	Independent (*n* = 111), mean (s.d.) or *n* (%)	Total (*N* = 226), mean (s.d.) or *n* (%)
Age (years) median (IQR)	30 (24–41)	28 (22–38)	29 (23–39)
Age ≥25 years	81 (70)	70 (63)	151 (67)
Male	80 (70)	75 (68)	155 (69)
Ethnicity			
White	90 (78)	91 (82)	181 (80)
Black	11 (10)	12 (11)	23 (10)
Asian	12 (10)	5 (5)	17 (8)
Other	2 (2)	3 (3)	5 (2)
Short Adaptive Behaviour Scale (proxy measure of level of intellectual disability)	50 (25)	56 (22)	52 (24)
Neurodevelopmental disorder			
Autism spectrum disorder or ADHD	72 (63)	72 (65)	144 (64)
Neither	33 (29)	33 (30)	66 (29)
Both	10 (9)	6 (5)	16 (7)
Aetiology of intellectual disabilities			
Unknown	95 (83)	86 (77)	181 (80)
Other	12 (11)	20 (18)	32 (14)
Down or fragile X syndrome	7 (6)	5 (5)	12 (5)
Physical health problems			
Mobility problems	45 (39)	40 (36)	85 (38)
Visual or hearing impairment*	60 (52)	75 (68)	135 (60)
Epilepsy	33 (29)	25 (23)	58 (26)
Incontinence	41 (36)	35 (32)	76 (34)
Other physical health problem(s)	33 (29)	35 (32)	68 (30)
Marital status			
Single	113 (98)	111 (100)	224 (99)
Married	2 (2)	−	2 (1)
Divorced	−	−	
Living situation			
Alone or with partner with/without children	23 (20)	22 (20)	45 (20)
With parents/relatives	34 (30)	24 (22)	58 (26)
Other (e.g. sheltered accommodation, supported living)	58 (50)	65 (59)	123 (54)
Accommodation			
Time in current address (months) median (IQR)	48 (12–144)	36 (11–146)	47 (12–144)
Family home	38 (33)	30 (27)	68 (30)
Supported living	34 (30)	47 (42)	81 (36)
Residential	33 (29)	27 (24)	60 (27)
Independent	9 (8)	7 (6)	16 (7)
Less than 6 months in current accommodation	13 (11)	19 (17)	32 (14)
Main source of income			
Salary/wage	−	1 (1)	1 (0.4)
Family support	35 (30)	27 (24)	62 (27)
State benefits	115 (100)	111 (100)	226 (100)
Occupational and activities status			
None	67 (58)	73 (66)	140 (62)
Full/part-time employment	2 (2)	2 (2)	4 (2)
Education, day centre, looking after home, other*	52 (45)	35 (32)	87 (38)
Voluntary work	6 (5)	6 (5)	12 (5)

IQR, interquartile range; ADHD, attention-deficit hyperactivity disorder.

* *P*<0.05.

### Primary outcome

Baseline mean total ABC-C scores were similar between IST models (enhanced 64, s.d. = 34; independent 62, s.d. = 32) (Supplementary Table 1 available at https://doi.org/10.1192/bjo.2023.74/). The mean ABC-C scores were lower at 9 months for both IST models (enhanced 56, s.d. = 34; independent 49, s.d. = 32) (Supplementary Table 1). Both unadjusted and adjusted analyses found no statistically significant difference in total ABC-C score change between IST models at 9 months (adjusted *β* = 4.27; 95% CI –6.34 to 14.87) (Table 2). The only predictors of missingness were physical health conditions.

**Table 2:** Change in clinical outcomes of intensive support teams at 9 months in terms of independent intensive support teams

	Difference between IST model			
	Unadjusted		Adjusted	
Outcome	Estimate	95% CI	Estimate	95% CI
Primary outcome (*β-*coefficient)				
Change in ABC-C total	3.08	−7.32 to 13.48	4.27	−6.34 to 14.87
Adjusted for time between baseline and 9-month data collection	2.75	−7.56 to 13.06	3.62	−6.99 to 14.22
Change in ABC-C, irritability	1.14	−2.41 to 4.69	2.09	−1.67 to 5.86
Change in ABC-C, lethargy	1.40	−1.77 to 4.58	1.31	−1.82 to 4.44
Change in ABC-C, stereotypic behaviour	−0.05	−1.44 to 1.34	0.69	−0.77 to 2.14
Change in ABC-C, hyperactivity	−0.07	−3.54 to 3.39	−0.11	−3.68 to 3.45
Change in ABC-C, inappropriate speech	0.83	−0.10 to 1.77	0.37	−0.86 to 1.60
Secondary outcomes (unadjusted)				
PAS-ADD organic condition (odds ratio)	1.09	0.39–3.02		
PAS-ADD affective or neurotic disorder (odds ratio)	0.91	0.32–2.59		
PAS-ADD psychotic disorder (odds ratio)	1.08	0.21–5.50		
Change in TAG score (*β-*coefficient)	1.11	−0.35 to 2.57	1.12	−0.44 to 2.68
Change in QOL-Q score (*β*-coefficient)	−0.75	−3.62 to 2.11	−2.63	−5.65 to 0.40

ABC-C, Aberrant Behaviour Checklist-Community version 2; PAS-ADD, Psychiatric Assessment Schedule for Adults with Developmental Disabilities Checklist; TAG, Threshold Assessment Grid; QOL-Q, Quality of Life Questionnaire.

### Secondary outcomes

No statistically significant differences were found in any of the secondary outcomes between IST models at 9 months (Table 2, Supplementary Table 1).

### Medication use

The mean number of medications prescribed at baseline was the same for both models (*n* = 5). At follow-up, the mean number was slightly reduced in the independent model (four for the independent versus five for the enhanced). At baseline, psychotropic medication was prescribed at similar proportions in both models: antipsychotic (enhanced 17% *v*. independent 18%) and other psychotropic (enhanced 35% *v*. independent 30%). The relative proportions of prescribed antipsychotics and other psychotropics did not change at follow-up: antipsychotics (enhanced 18% *v*. independent 20%) and other psychotropics (enhanced 31% *v*. independent 32%). Of those who were on psychotropics, over two-thirds were prescribed more than one medication in both models.

### Psychiatric hospital admissions and change in accommodation

Over the study duration, eight participants in the enhanced model and 11 participants in the independent were admitted to a psychiatric unit as a result of a mental health crisis. Nine (4%) participants moved accommodation during the study period. All but one participant lived in supported living or residential provision.

### Cost evaluation

The average annual cost of teams in the enhanced model was £612 612 (£4980 per case), whereas the average annual cost of a team in the independent model was £647 812 (£10 122 per case) (Supplementary Table 2).

### Service use

From an NHS/Personal Social Services perspective, the mean total cost over 9 months was £22 915.6 for the independent model and £19 037.6 for the enhanced model; the adjusted mean difference in costs was not statistically significant (£446.55; 95% CI −5637.60 to £7519.30).

From a societal perspective the mean total cost over 9 months was £31 850.8 for the independent model and £29 852.8 for the enhanced. The adjusted mean difference in costs was not statistically significant (−£855.80; 95% CI −£8342.54 to £6059.69).

The mean use of in-patient, out-patient and day patient health services over the 9-month follow-up period are reported in Supplementary Table 3. Duration of in-patient stay, out-patient attendances, day hospital contacts and emergency (accident and emergency department) attendance, were broadly similar for both models. Notably, participants in the independent model spent longer, on average, as in-patients than those in the enhanced model (mean 8.63 days (s.d. 39.98) *v*. 5.26 days (s.d. 28.10). Participants in the independent model had, on average, more contacts with their general practitioner than participants in the enhanced model (mean 4.88 days (s.d. 14.20) *v*. 3.47 days (s.d. 3.96) attendances), although these were not statistically significant.

### Cost-effectiveness

There are no statistically significant differences in QALYS in any of the comparisons of the service models at 9 months (Supplementary Table 4). Results from the regression analysis using the two outcomes of total ABC-C score and QALYs are summarised with incremental cost-effectiveness ratios in Supplementary Table 5.

Probability estimates were plotted for a range of implicit monetary values attached to improvements in total ABC-C score and QALY gain over 9 months under an NHS and societal perspective, in turn (Supplementary Figs 1−4).

The independent model had a low likelihood (approximately 50%) of being more cost-effective than the enhanced model if decision makers were not willing to pay anything for a unit improvement in the total ABC-C score. The likelihood of cost-effectiveness rose to 70% if willingness to pay for an improvement in total ABC-C score rose to £1000. Under a broader perspective, which includes cost of unpaid support, the probability of the independent model being cost-effective when compared with the enhanced model at the standard NICE-preferred willingness-to-pay levels of £20 000–30 000 per QALY, was 52%. It is therefore unlikely that there are any economic gains from choosing one model of care over another. Controlling for factors contributing to missing data in health and social care costs in sensitivity analyses did not alter the findings of the main analyses.

## Discussion

The study showed that both IST models currently in operation in England were associated with reduction in behaviours that challenge at 9 months follow-up, with comparable costs.

The participants in both IST models appeared to score at levels on risk similar to those who were admitted to hospital in a previous study of an in-patient psychiatric ward.^20^ Another study of predictors on in-patient admission that used routine clinical data did not include a measure of risk.^30^

Over 28 months, during which time we enrolled and assessed participants at two time points, there were 19 admissions, averaging fewer than one admission per month across all services that took part. However, this is at odds with the monthly data release by the NHS Digital Learning Disability Services Monthly Statistics at the end of 2020^31^ (closest date to the end of the final participant 9-month follow-up), which shows that 90 were admitted to an in-patient unit that month. When we started the participant enrolment in the autumn of 2018, there were 125 first or readmissions. There are several considerations about the interpretation of this information. First, NHS Digital reports on both people with intellectual disabilities and people with autism, so it is possible that the figures are inflated because of the diversity of the patient cohort. Second, our reporting is based on 21 services whereas NHS Digital collects data from a greater number of services.

Most recently, Dodd et al^32^ reported on a new model of integrating intensive support service with an in-patient unit for adults with complex needs, including behaviours that challenge, at risk of admission. The new remodelled service appears to have had increasing efficacy in preventing admissions in 90% of referrals in 2020; this was up from about 60% of those referred prior to remodelling. Placement of patients was maintained in up to 80% of cases. However, although duration of in-patient care was reduced from 18 months to less than 12 months as a result of the intensive support, delayed discharges remained mostly as a consequence of lack of suitable accommodation. Bohen and Woodrow^33^ and Mottershead and Woodrow^34^ published initial findings for the Dynamic Support Database clinical support tool. Such work, although in its early stages, is promising and points toward prevention and intervention strategies that could contribute to the refinement of the specification of ISTs. The recent publication of the action plan^35^ relating to ‘Building the Right Support’^3^ does not make reference to ISTs or how they fit into current practice, especially regarding the stated intent to ‘close inpatient facilities for people with learning disabilities and/or autism who display behaviour that challenges’. Therefore, there is still no specific guidance as to how ISTs are to be implemented across England. Other projects relating to the Transforming Care programme have yet to report findings and do not include examination of community support, intensive or routine (e.g. https://www.birmingham.ac.uk/schools/social-policy/departments/social-work-social-care/research/why-are-we-stuck-in-hospital.aspx).

Finally, the interplay between multiple medications including psychotropics and the display of behaviour that challenges is a complex one. Apart from the interactions between different pharmacological agents, changes in psychotropic medication regimens can also affect behaviour, as is well recognised.^35^ Future research should consider a more granular approach to medication administration and prescribing, given the significant numbers of participants on psychotropics and other drugs.

The setting of ISTs (rural or urban areas) has not been analysed in this study because literature in other populations indicates that individual demographic and clinical characteristics, rather than location, are more likely to affect adverse outcomes such as hospital admissions.^36^

The only studies including a health economic evaluation are by Iemmi et al,^37^ who reported on costs of one IST delivering positive behaviour support to five patients associated with improved outcomes at a total cost of health and social care services of £2,296 per week. Hassiotis et al^38^ highlighted that a specialist behaviour team maybe cost neutral when compared with treatment as usual.

The health economic evaluation of this multicentre study showed that the service costs of enhanced ISTs are not significantly different from the independent ISTs, and neither are the health-related quality-of-life gains. However, as the economic burden of behaviours that challenge remains substantial,^10,14,39^ it is likely that costs could be offset by clinical improvements associated with IST care. Cost per case would range between £4980 and £10 122 based on number of referrals per model, enhanced and independent, respectively. However, the magnitude of that change would have to exceed the unit improvement in the total ABC-C score to be considered as clinically significant, and when all costs of health and social care are taken into account the cost differences between models are not significant. Previous research indicates that experts by experience expect larger differences as a result of interventions to be clinically meaningful than those reported in existing publications.^40^

### Strengths and limitations

This study is the first, to our knowledge, to have systematically evaluated IST models in England. It was fully powered with very good retention of participants (<10% attrition), the COVID-19 pandemic notwithstanding. ISTs were representative of such services in England and the study participants representative of the population on IST case-loads, which minimise the risk of bias. The findings from this study are highly relevant to the support of very vulnerable individuals with intellectual disabilities in the community, and potentially applicable to other UK countries where they seek to establish similar approaches to the acute or preventive management of behaviours that challenge.

The study also has limitations. First, responses might be subject to respondent social desirability bias. Second, this was not a randomised controlled trial, so there may have been differences between groups that we were unable to measure and adjust for in the analyses. Third, the turnover of paid carers may have affected the reporting of behaviours that challenge if the carer had not known the person with intellectual disabilities for long enough. Fourth, the lack of statistical significance in clinical outcomes between models may be an indication that adults who are referred during a crisis will recover in the short to medium term as behaviour that challenges is a remitting/relapsing condition (regression to the mean).^4^ Fifth, as we did not recruit participants at the point of referral to the IST, we must be cautious about the change that was achieved, as it has not taken into account any improvements made before study entry. Sixth, there may have been some effects from the COVID-19 pandemic, as 131 follow-up interviews were conducted from March 2020 to January 2021, but we were unable to fully adjust for it. For example, the pandemic may have exacerbated behaviours that challenge or affected the patterns and intensity of service use in both models. This is especially important, given the current disproportionate impact of COVID-19 on people with intellectual disabilities, including higher death rates.^41^ Finally, we did not collect process outcomes such as Care and Treatment Reviews (CTRs) completed by the teams, although it appears that almost half of those admitted had a CTR within 6 months of admission. Therefore, it is likely that CTRs may not be the sole reason for failing to prevent an admission.

In conclusion, our findings indicate that commissioners can choose which IST model is relevant to their localities, but also that there is a need to further investigate the critical ingredients of effective IST care and understand how best ISTs may work with and fit into the wider mental health service system. This information should be incorporated within the action plans about the right community support for adults with intellectual disabilities who display behaviours that challenge.

## Data Availability

Anonymised data of the study are available from A.H. on request and subject to internal review of proposals.

## Supplementary material

For supplementary material accompanying this paper visit http://doi.org/10.1192/bjo.2023.74. click here to view supplementary material

## References

[1] Bowring DL, Totsika V, Hastings RP, Toogood S, McMahon M. Prevalence of psychotropic medication use and association with challenging behaviour in adults with an intellectual disability. a total population study. J Intellect Disabil Res 2017; 61(6): 604–17. 28090687 10.1111/jir.12359

[2] National Institute for Health and Care Excellence (NICE). Learning Disabilities and Behaviour that Challenges: Service Design and Delivery. NICE Guideline [NG93]. NICE, 2018 (https://www.nice.org.uk/guidance/ng93/resources/learning-disabilities-and-behaviour-that-challenges-service-design-and-delivery-pdf-1837753480645).

[3] NHS England. Supporting People with a Learning Disability and/or Autism Who Have a Mental Health Condition or Display Behaviour that Challenges. *NHS England*, 2015 (https://www.england.nhs.uk/wp-content/uploads/2015/07/ld-draft-serv-mod.pdf).

[4] Cooper SA, Smiley E, Jackson A, Finlayson J, Allan L, et al. Adults with intellectual disabilities: prevalence, incidence and remission of aggressive behaviour, and related factors. J Intellect Disabil Res 2009; 53(3): 200–16. 18444987 10.1111/j.1365-2788.2008.01060.x

[5] Durbin A, Balogh R, Lin E, Wilton AS, Lunsky Y. Emergency department use: common presenting issues and continuity of care for individuals with and without intellectual and developmental disabilities. J Autism Dev Disord 2018; 48(10): 3542–50. 29923146 10.1007/s10803-018-3615-9

[6] Durbin A, Balogh R, Lin E, Wilton AS, Selick A, Dobranowski KM, et al. Repeat emergency department visits for individuals with intellectual and developmental disabilities and psychiatric disorders. Am J Intellect Dev Disabil 2019; 124(3): 206–19. 31026200 10.1352/1944-7558-124.3.206

[7] Lin E, Balogh R, Chung H, Dobranowski K, Durbin A, Volpe T, et al. Looking across health and healthcare outcomes for people with intellectual and developmental disabilities and psychiatric disorders: population-based longitudinal study. Br J Psychiatry 2021; 218(1): 51–7. 33161927 10.1192/bjp.2020.202

[8] Taggart L, McMillan R, Lawson A. Predictors of hospital admission for women with learning disabilities and psychiatric disorders compared with women maintained in community settings. Adv Ment Heal Learn Disabil 2009; 3(1): 30–41.

[9] Painter J, Ingham B, Trevithick L, Hastings RP, Roy A. Correlates for the risk of specialist ID hospital admission for people with intellectual disabilities: development of the LDNAT inpatient index. Tizard Learn Disabil Rev 2018; 23(1): 42–50.

[10] Department of Health. Transforming Care: A National Response to Winterbourne View Hospital. Department of Health, 2012 (https://www.gov.uk/government/uploads/system/uploads/attachment_data/file/213215/final-report.pdf).

[11] Community and Mental Health Team, Health and Social Care Information Centre. *Learning Disability Census Report*. Health and Social Care Information Centre, 2015 (https://files.digital.nhs.uk/publicationimport/pub19xxx/pub19428/ld-census-initial-sep15-rep.pdf).

[12] Davison S, Mcgill P, Baker P, Allen D. A national UK survey of periopatetic support teams for children and adults with intellectual and developmental disability who display challenging behaviour. Int J Posit Behav Support 2015; 5(1): 26–33.

[13] Hassiotis A. Community mental health services for individuals with intellectual disabilities. Dis Manag Heal Outcomes 2002; 10: 409–17.

[14] National Institute for Health and Care Excellence (NICE). Challenging Behaviour and Learning Disabilities: Prevention and Interventions for People with Learning Disabilities whose Behaviour Challenges. NICE Guideline [NG11]. NICE, 2015 (https://www.nice.org.uk/guidance/ng11/). 26180881

[15] Hassiotis A, Walsh A, Budgett J, Harrison I, Jones R, Morant N, et al. Intensive support for adults with intellectual disability and behaviours that challenge: a survey of provision and service typologies in England. BJPsych Open 2020; 6(2): e20. 32043438 10.1192/bjo.2020.2 PMC7176866

[16] Aman MG, Singh NN, Stewart AW, Field CJ. The Aberrant Behavior Checklist: a behavior rating scale for the assessment of treatment effects. Am J Ment Defic 1985; 89(5): 485–91. 3993694

[17] Prosser H, Moss S, Costello H, Simpson N, Patel P, Rowe S. Reliability and validity of the Mini PAS-ADD for assessing psychiatric disorders in adults with intellectual disability. J Intellect Disabil Res 1998; 42: 264–72. 9786440 10.1046/j.1365-2788.1998.00146.x

[18] Slade M, Powell R, Rosen A, Strathdee G. Threshold Assessment Grid (TAG): the development of a valid and brief scale to assess the severity of mental illness. Soc Psychiatry Psychiatr Epidemiol 2000; 35(2): 78–85. 10784370 10.1007/s001270050011

[19] Shalock RL, Keith KD. Quality of Life Questionnaire Manual. IDS Publishing Corporation, 1993.

[20] Hall I, Parkes C, Samuels S, Hassiotis A. Working across boundaries: clinical outcomes for an integrated mental health service for people with intellectual disabilities. J Intellect Disabil Res 2006; 50(8): 598–607. 16867067 10.1111/j.1365-2788.2006.00821.x

[21] Herdman M, Gudex C, Lloyd A, Janssen M, Kind P, Parkin P, et al. Development and preliminary testing of the new five-level version of EQ-5D (EQ-5D-5L). Qual Life Res 2011; 20(10): 1727–36. 21479777 10.1007/s11136-011-9903-x PMC3220807

[22] Beecham J. Collecting and estimating costs. In The Economic Evaluation of Mental Health Care (ed M Knapp): 157–74. Arena, 1995.

[23] Hatton C, Emerson E, Robertson J, Gregory N, Kessissoglou S, Perry J, et al. The Adaptive Behavior Scale-Residential and Community (part I): towards the development of a short form. Res Dev Disabil 2001; 22(4): 273–88. 11523952 10.1016/s0891-4222(01)00072-5

[24] Hassiotis A, Poppe M, Strydom A, Vickerstaff V, Hall IS, Crabtree J, et al. Clinical outcomes of staff training in positive behaviour support to reduce challenging behaviour in adults with intellectual disability: cluster randomised controlled trial. Br J Psychiatry 2018; 212(3): 161–8. 29436314 10.1192/bjp.2017.34

[25] StataCorp. Stata Statistical Software: Release 16. StataCorp LLC, 2019 (https://www.stata.com).

[26] Brouwer W, Rutten F, Koopmanschap M. Costing in economic evaluation. In Economic Evaluation in Health Care (eds M Drummond, A McGuire): 68–93. Oxford University Press, 2001.

[27] Stinnett AA, Mullahy J. Net health benefits: a new framework for the analysis of uncertainty in cost-effectiveness analysis. Med Decis Mak 1998; 18(suppl 2): 68–80. 10.1177/0272989X98018002S09 9566468

[28] van Hout B, Janssen M, Feng Y, Kohlmann T, Busschbach J, Golicki D, et al. Interim scoring for the EQ-5D-5L: mapping the EQ-5D-5L to EQ-5D-3L value sets. Value Heal 2012; 15(5): 708–15. 10.1016/j.jval.2012.02.008 22867780

[29] Fenwick E, Byford S. A guide to cost-effectiveness acceptability curves. Br J Psychiatry 2003; 187(2): 106–108. 10.1192/bjp.187.2.106 16055820

[30] Sheehan R, Mutch J, Marston L, Osborn D, Hassiotis A. Risk factors for in-patient admission among adults with intellectual disability and autism: investigation of electronic clinical records. BJPsych Open 2020; 7(1): e5. 33256877 10.1192/bjo.2020.135 PMC7791557

[31] NHS Digital. Learning Disability Services Monthly Statistics. NHS Digital, 2020 (https://digital.nhs.uk/data-and-information/publications/statistical/learning-disability-services-statistics/learningdisability-services-monthly-statistics-at-november-2020-mhsds-september-2020-final).

[32] Dodd K, Laute V, Daniel S. The development and evaluation of an integrated intensive support service. Adv Ment Health Intellect Disabil 2022; 16: 1–7.

[33] Bohen F, Woodrow C. Dynamic support database clinical support tool: inter-rater reliability. Adv Ment Heal Intellect Disabil 2020; 14(2): 25–32.

[34] Mottershead T, Woodrow C. Practicality, utility and face-validity of the dynamic support database. Adv Ment Heal Intellect Disabil 2019; 13(5): 228–36.

[35] Sheehan R, Hassiotis A. Reduction or discontinuation of antipsychotics for challenging behaviour in adults with intellectual disability: a systematic review. Lancet Psychiatry 2017; 4(3): 238–56. 27838214 10.1016/S2215-0366(16)30191-2

[36] Osborn D, Lamb D, Canaway A, Davidson M, Favarato G, Pinfold V, et al. Acute day units in non-residential settings for people in mental health crisis: the AD-CARE mixed-methods study. Health Serv Deliv Res 2021; 9(18): 1–122. 34609810

[37] Iemmi V, Knapp M, Saville M, McWade P, McLennan K, Toogood S. Positive behavioural support for adults with intellectual disabilities and behaviour that challenges: an initial exploration of the economic case. Int J Posit Behav Support 2015; 5(1): 16–25.

[38] Hassiotis A, Robotham D, Canagasabey A, Romeo R, Langridge D, Blizard R, et al. Randomized, single-blind, controlled trial of a specialist behavior therapy team for challenging behavior in adults with intellectual disabilities. Am J Psychiatry 2009; 166(11): 1278–85. 19687128 10.1176/appi.ajp.2009.08111747

[39] Hassiotis A, Parkes C, Jones L, Fitzgerald B, Romeo R. Individual characteristics and service expenditure on challenging behaviour for adults with intellectual disabilities. J Appl Res Intellect Disabil 2008; 21(5): 438–45.

[40] Hassiotis A, Melville C, Jahoda A, Strydom A, Cooper SA, Taggart L, et al. Estimation of the minimal clinically important difference on the Aberrant Behaviour Checklist–Irritability (ABC-I) for people with intellectual disabilities who display aggressive challenging behaviour: a triangulated approach. Res Dev Disabil 2022; 124: 104202. 35248813 10.1016/j.ridd.2022.104202

[41] NHS England. *COVID-19 Deaths of Patients with a Learning Disability Notified to LeDeR*. NHS England, 2022 (https://www.england.nhs.uk/publication/covid-19-deaths-of-patients-with-a-learning-disability-notified-to-leder/).

